# Hydrate of Chloral in Labor

**Published:** 1876

**Authors:** C. A. Prentiss

**Affiliations:** Greenfield, Missouri


### HYDRATE OF CHLORAL IN LABOR.

By C. A. Prentiss, M.D., of Greenfield, Missouri.

The article of Dr. Chapman (in the July number of this *Journal*) prompts me to add my evidence in favor of chloral in parturition.

It is a grave error to expect that chloral will relieve all the pain of labor when administered in such doses as will, at all times, be safe. There is an amount of false pain accompanying labor, which is very tormenting to patients, and not in any degree to their advantage. To relieve this was my object in giving chloral in the first instance; but after trial in many cases, I can report the following effects which I have observed;

1. A quiet and tranquil state of mind.
2. Abridgment of the duration, and modification of the severity of each pain.
3. Pleasant and refreshing sleep in the interval of pain.
4. Relaxation of the os and consequent abridgment of the duration of labor.

I have attended patients who were very nervous and excited by their forebodings of a serious termination, who in thirty minutes abandoned all such thoughts, and indulged in brighter and better hopes.

Chloral modifies the severity of the pains, and limits their duration to the time the uterus is actually in contraction. Every practitioner has, no doubt, observed many cases in which the pain continues after the active contraction ceases, and the os softens. This is what I have characterized as "false pain."

I have frequently witnessed patients very much exhausted by the suffering, in some instances, of protracted labor, refreshed and their strength restored by pleasant naps in the intervals of pain.

I was called to attend a lady who had been in labor over twelve hours. The pains were frequent and active, and the os dilated to about one inch in diameter, very firm and rigid. The old midwife in attendance informed me that no progress had been made in the last six or eight hours. I began the administration of chloral at once, and attended very closely to the case. In about forty minutes the rigidity began to give way, and in one hour more, labor was completed. This is only one case, to be sure, but to cite more would only be to repeat the history of this.

The distressing after-pains are also modified, and may be further relieved by small doses of paregoric.

My plan of giving the chloral is as follows: commence in the first stage with gr. v every fifteen minutes, until gr. xx or xxx are taken, more to be given in four to six hours if labor continue and the remedy be indicated, but not to the extent of producing anæsthesia. This amount I have found to be sufficient to prodnce all the effects I deem desirable. Some practitioners have recommended its use to the extent of inducing anæsthesia, but this I consider unsafe, unnecessary, and unjudicious.—*American Journal of the Medical Sciences.*

